# Nematode communities indicate diverse soil functioning across a fog gradient in the Namib Desert gravel plains

**DOI:** 10.1002/ece3.9013

**Published:** 2022-06-17

**Authors:** Amy M. Treonis, Eugene Marais, Gillian Maggs‐Kölling

**Affiliations:** ^1^ Department of Biology University of Richmond Richmond Virginia USA; ^2^ Gobabeb‐Namib Research Institute Erongo Namibia

**Keywords:** anhydrobiosis, biological soil crusts, desert soil, fog, nematode diversity, soil biodiversity

## Abstract

Soil nematodes are fundamentally aquatic animals, requiring water to move, feed, and reproduce. Nonetheless, they are ubiquitous in desert soils because they can enter an anhydrobiotic state that allows them to persist when water is biologically unavailable. In the hyper‐arid Namib Desert of Namibia, rain is rare, but fog routinely moves inland from the coast and supports plant and animal life. Very little is understood about how this fog may affect soil organisms. We investigated the role of fog moisture in the ecology of free‐living, soil nematodes across an 87‐km fog gradient in the gravel plains of the Namib Desert. We found that nematodes emerged from anhydrobiosis and became active during a fog event, suggesting that they can utilize fog moisture to survive. Nematode abundance did not differ significantly across the fog gradient and was similar under shrubs and in interplant spaces. Interplant soils harbor biological soil crusts that may sustain nematode communities. As fog declined along the gradient, nematode diversity increased in interplant soils. In areas where fog is rare, sporadic rainfall events can stimulate the germination and growth of desert ephemerals that may have a lasting effect on nematode diversity. In a 30‐day incubation experiment, nematode abundance increased when soils were amended with water and organic matter. However, these responses were not evident in field samples, which show no correlations among nematode abundance, location in the fog gradient, and soil organic matter content. Soil nematodes are found throughout the Namib Desert gravel plains under a variety of conditions. Although shown to be moisture‐ and organic matter‐limited and able to use moisture from the fog for activity, variation in fog frequency and soil organic matter across this unique ecosystem may be biologically irrelevant to soil nematodes in situ.

## Graphical abstract

In the Namib Desert gravel plains, soil nematode communities vary along a moisture gradient that stretches through the coastal fog‐fed zone into areas relying mainly on rainfall. Soil nematodes are adapted to respond to moisture from fog by emerging from anhydrobiosis and regaining activity. Nematode diversity was higher in the rainfall zone, however, likely due to the legacy effects of ephemeral vegetation that grows in response to rare rain events.

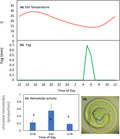

## 1 INTRODUCTION

“The Gravel Flats of the Outer Namib are of almost unbelievable monotony. Over large areas, their local relief can be measured in increments of inches per acre”. (Logan, 1960).

**FIGURE 1:**
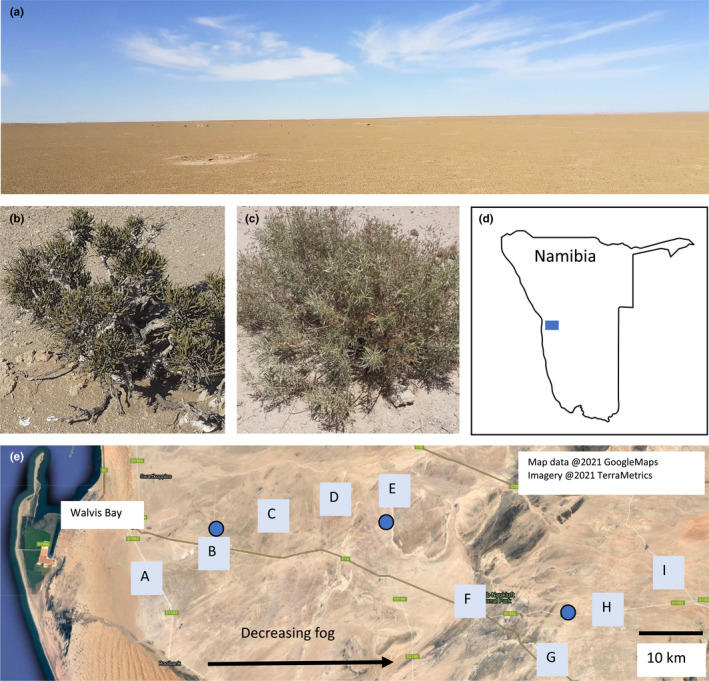
(a) Representative landscape of the Namib Desert gravel plains, (b) *Arthraerua leubnitziae* (pencilbush), (c) *Pechuel‐loeschea leubnitziae* (wild sage), (d) map of Namibia with the location of field sites in the blue rectangle, (e) arrangement of field sites (gray squares) moving inward from the coast along a gradient of decreasing fog. Blue circles represent meteorological stations (from left to right, Kleinberg, Marble Koppie, and Garnet Koppie). The line extending eastward from Walvis Bay is the C14 road, the main route through the region

The Namib Desert is a narrow, hot desert that extends 2000 km along the western coast of southern Africa, from Angola, through Namibia, and into South Africa. Aridity in coastal Namibia is maintained by the Benguela Current, which brings cold water to the area and limits the formation of rainfall‐producing fronts. While the Namib Desert is widely known for its soaring, red‐orange dunes (i.e., the Namib “Sand Sea,” a UNESCO World Heritage site), much of the Namib Desert is composed of gravel plains with little topographical relief. Nearly barren of vegetation, the gravel plains offer minimalism (and monotony) on a grand scale (Figure 1a).

The Namib Desert is among a small group of global deserts that receive moisture from the fog as well as sporadic rainfall (~5–100 mm precipitation annually, Eckardt et al., 2013). The distribution of fog and rain varies considerably over time and space. Cold fogs and mists can extend far inland, but their frequency decreases with distance from the coast, while rain inputs tend to increase (Eckardt et al., 2013; Spirig et al., 2019). Fog is rare at 60 km from the coastline and beyond (Kaseke et al., 2018), but at 33 km, it may be present on up to 87 days of the year (Mitchell et al., 2020), making fog a relatively reliable source of moisture in parts of the gravel plains. The role of fog in the ecology of many Namib Desert species, including beetles, lizards, grasses, and shrubs has been well‐documented (Jacobson et al., 2015; Mitchell et al., 2020; Wang et al., 2019). Many organisms have evolved morphological and behavioral adaptations to capture moisture from fog (Mitchell et al., 2020). Less is known about the possible use of fog by belowground biota, although fog‐induced increases in soil water potential have been measured in gravel plains soils (Li et al., 2018; Wang et al., 2019).

Namib Desert gravel plains soils contain many of the invertebrate taxa found in soils globally, including microarthropods such as collembola (Collins et al., 2019) and mites (André et al., 1997), but little is known about soil nematodes. Nematode diversity and abundance have been studied in soils under *Acanthosicyos horridus* (!nara melon shrubs) in the Namib Desert sand dunes (Marais et al., 2020), and some nematode species have been described that were isolated from soil associated with *Welwitschia mirabilis* plants in the gravel plains (Rashid et al., 1990a, 1990b; Rashid & Heyns, 1990a, 1990b). However, nematode distribution and abundance in the gravel plains have not been systematically investigated, and whether the activity, abundance, and diversity of these animals are connected to fog is unknown. Nematodes reside within water films on the surfaces of soil particles (Yeates, 2004). In the absence of water, nematodes become quiescent, entering an ametabolic state known as anhydrobiosis (Demeure et al., 1979; Treonis et al., 2000). When nematodes are anhydrobiotic, they coil their bodies, and they cannot feed, move, or reproduce (Crowe et al., 1992). Prior research has shown that soil nematodes in desert ecosystems rapidly become active in response to rainfall (Whitford et al., 1981) and melting snowfall (Treonis et al., 2000). It is unknown whether nematodes may be similarly sensitive to fog events.

Soil nematode communities rely on vegetation as either a direct source of energy (e.g., plant‐parasitic taxa) or indirectly through the detrital food web (bacterivores, fungivores, etc.). In deserts, where plant productivity is limited by the extreme environment, nematode abundance usually is highest in association with perennial plants, such as shrubs (Freckman & Mankau, 1977; Pen‐Mouratov et al., 2003; Treonis et al., 2019). However, biological soil crusts, found in interplant spaces in arid ecosystems, also can support nematode communities (Darby et al., 2007). The periodic germination and growth of desert ephemerals also have been found to increase soil nematode abundance (Treonis et al., 2019).

We studied soil nematode communities in the gravel plains of the Namib Desert in a series of experiments. The purpose of this work was to determine how the fog gradient may impact the abundance and diversity of soil nematodes. First, we conducted a survey of nematode abundance and diversity across the gradient in shrub and interplant soils. We predicted that nematode communities would be more abundant and diverse under shrubs and where fog precipitation is highest. Second, we investigated the degree to which nematodes are limited by moisture and energy availability in a laboratory incubation experiment in which we amended soils with water and/or organic matter. We predicted that these would be co‐limiting factors for nematode abundance in gravel plains soils. Finally, we studied the activity of soil nematodes in situ during a fog event. We predicted that nematodes in gravel plains soils could utilize fog moisture to emerge from anhydrobiosis.

## 2 METHODS

### 2.1 Study site

This work was conducted in the central region of the Namib Desert, in the gravel plains between the Kuiseb and Swakop Rivers (Figure 1e). Soils in the gravel plains are classified as Petric Gypsisols (Sites A–E) or Petric Calcisols (Sites F–I). Meteorological data were obtained from the Southern African Science Service Centre for Climate Change and Adaptive Land Management (SASSCAL, http://www.sasscalweathernet.org/), which curates data from the Gobabeb‐Namib Research Institute's meteorological stations within the study area. A 4‐year time span (2017–2020) was chosen for analysis because it was most relevant to (and overlapped with) the study period, but more detailed analyses of Namib Desert weather can be found in Spirig et al. (2019), Kaseke et al. (2017), and Eckardt et al. (2013).

### 2.2 Gravel plains nematode communities across the fog gradient

**FIGURE 2:**
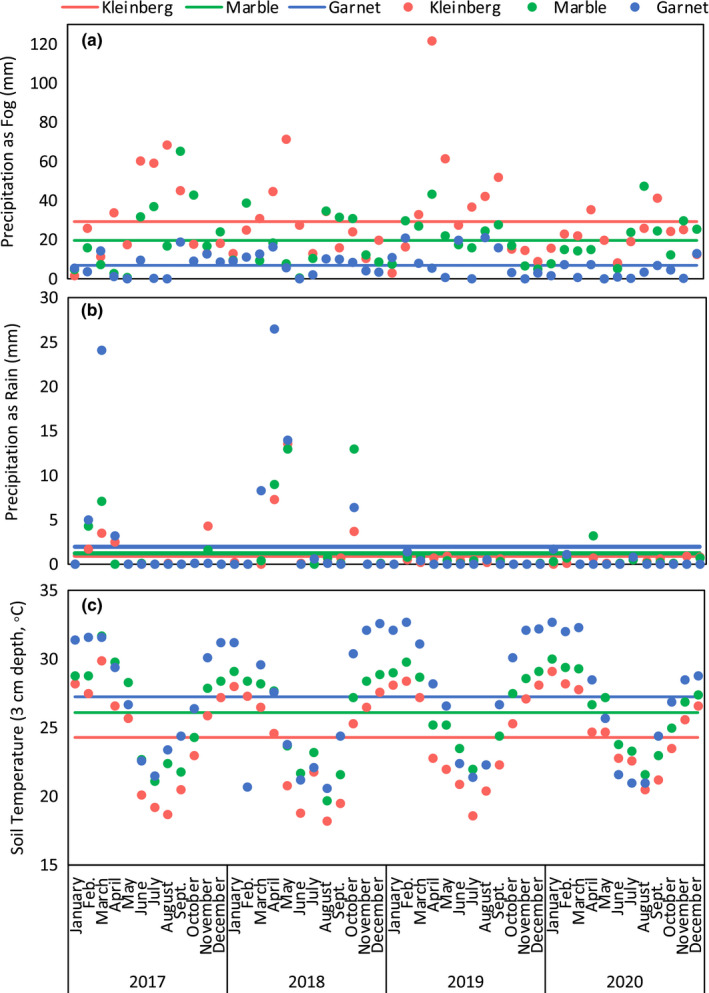
Meteorological data for the 4‐year period preceding and encompassing this study (2017–2020) from meteorological stations located near sampled sites in the gravel plains, with Kleinberg located closest to the coast and Garnet Koppie the farthest. (a) Precipitation as fog, (b) precipitation as rain, and (c) soil temperature (3‐cm depth). Points represent monthly totals for precipitation or the monthly average for temperature. Horizontal lines represent the averages for these data across the 4‐year time period

Soils were collected from nine sites (A–I) beginning 18 km from the coast, near Walvis Bay, and extending inland 87 km (Figure 1e, Table 1). Soils were collected at 10‐km intervals moving inland on 14 May 2020 (sites A–E) and 19 September 2020 (sites F–I). Ideally, all sites would have been sampled at approximately the same time, but this was not possible due to Covid‐19 lockdowns and travel restrictions in Namibia in 2020. However, there had been no significant rainfall in the region for at least 24 months (Figure 2), and the soils were very dry. Based on the prior experience of the authors studying nematodes in desert ecosystems, it is unlikely nematode communities at sites F–I underwent significant changes between May and September that could obfuscate comparison to soils from the earlier sampling date. Sites were selected to maximize distance from the main road through the region because traffic raises dust and imparts some degree of disturbance to adjacent soils. However, only existing off‐road tracks were used to access sites, which made it impossible to sample across the gradient in a straight line.

**TABLE 1:** Location, elevation, and shrub species at Namib Desert gravel plains sampling sites

Sampling site	Location (GPS coordinates in decimal degrees)	Elevation (meters above sea level)	Shrub species present
A	−23.0647, 14.6150	90	*Arthraerua leubnitziae*
B	−23.0223, 14.7198	179	*A. leubnitziae*
C	−22.9579, 14.8168	249	*A. leubnitziae*
D	−22.9431, 14.9144	348	*A. leubnitziae*
E	−22.9203, 15.0088	356	*A. leubnitziae*
F	−23.0971, 15.1156	577	*Pechuel‐loeschea leubnitziae*
G	−23.1813, 15.2628	669	*P. leubnitziae*
H	−23.1058, 15.3501	762	*P. leubnitziae*
I	−23.0518, 15.4520	877	*P. leubnitziae*

At the time of sampling, the interplant spaces were devoid of vascular plants, except for a few types of grass (*Stipagrostis* spp.) that were desiccated and grazed down to small stubs. At the sites nearest to the coast (A–C), biological soil crusts were visible in the interplant spaces. A single shrub species is not present across the sites, so two different plants were selected for sampling. At sites A–E, the most abundant shrub is the Namib Desert endemic, pencilbush *Arthraerua leubnitziae* (Kuntze) Schinz (family *Amaranthaceae*) (Figure 1b). At sites F–I, wild sage, *Pechuel‐loeschea leubnitziae* (Kuntze) O. Hoffm. (family *Asteraceae*) was sampled (Figure 1c).

At each site, three soil samples were collected from under shrubs and three from interplant spaces at least 1‐m away from shrubs. This level of replication was selected based on an analysis of the level of variation in nematode communities from preliminary samples that were collected from three sites along the fog gradient in March 2020. Samples (~700 g each) were collected to a depth of 5–10 cm with plastic scoops from multiple locations under the canopy of each shrub (leaf litter was scraped away first) or from several unique spots in the interplant area. Due to the dry, coarse nature of the soil, contaminating material that could contain nematodes was not retained on the scoops. We focused on the surface layer to study the nematodes most likely to be influenced by plant litter and fog. Some of the sites were underlain by a hard calcrete layer below 5 cm that limited sampling depth. Soils were sieved (2 mm) to remove rocks and transferred to sterile, air‐tight plastic bags. Rocks are removed prior to analyses because they are not considered to be soil material and are not porous to nematodes. The soil was not visibly attached to the rocks that were sieved from these very dry soils. Samples were stored in coolers at ambient temperature prior to analysis of nematode communities and soil properties.

We observed that sieving retained some biological crust material from interplant soils at sites A and B. To assess the potential of this crust material and other microhabitats for supporting nematodes, several other types of samples were analyzed. First, at site B samples were collected (29 July 2020) to investigate the distribution of nematodes with depth and within the biological crust material. Site B was selected because it receives fog routinely and is easily accessed via existing off‐road tracks. Four samples were collected from the top 0–1 cm of soil and sieved (2 mm) to separate rocks and large crust particles from the soil for analysis of nematodes. Four additional samples were collected from 1 to 5 cm. These soils were sieved to remove rocks. Second, three mixed‐species fruticose lichen samples (5–11 g fresh weight) were collected from site B and transferred into sterile, 50‐ml conical tubes. Third, at five sites (A–E), three quartz rock‐associated soil samples were collected to study hypolithic nematode communities. For each sample, several quartz rocks were gently lifted from the soil, and the soil adhering to the bottom of the rocks (~20 g) was scraped into conical tubes. Samples were stored in coolers at ambient temperature prior to analysis.

### 2.3 Soil analyses

Soil moisture was determined gravimetrically (24 h at 105°C, Gardner, 1986). Soil organic matter was measured as a loss on ignition (LOI, 360°C, 2 h, Nelson & Sommers, 1996). Suspensions of 10‐g air‐dried soil in 30‐ml deionized H_2_O were mixed and equilibrated for 30 min. Solution pH was measured with a Pocket Pro+ pH Tester (Hach, Loveland, CO), and using the same solution, electrical conductivity was measured as an indicator of salinity using a conductivity meter (Traceable®, Cole‐Parmer, Vernon Hills, IL) (Treonis et al., 2019).

### 2.4 Nematode community analyses

Nematodes were extracted over 72 h from 120 g soil using a Baermann funnel technique (60 g soil × 2 funnels per sample, combined when drawing off the solution, Baermann, 1917). The Baermann technique relies on nematode motility for extraction. Dead nematodes are not generally recovered. In desert soils, dead nematodes do not decompose quickly, and if other extraction techniques are used (e.g., density centrifugation), their presence may confound estimates of nematode density. We conducted an extraction method comparison (Baermann vs. density centrifugation, Jenkins, 1964) for a subset of Namib Desert soils (*n* = 12) and confirmed that both methods recovered a similar number of live nematodes. However, density centrifugation recovered more dead nematodes (an average of 68% of the nematodes recovered were dead). Over 98% of nematodes were alive following extraction with Baermann funnels in this trial, so we selected this method to use for experimental samples. After extraction, sample volume was reduced, and nematodes were fixed in a 5% hot:cold formalin solution. All nematode extractions from field samples were performed within 30 days of the collection of soils, which had been stored in airtight bags in coolers at ambient temperatures (10–23°C). Given the desiccated state of the soils, and the likely inactivity of nematodes, refrigeration was not necessary. Nematodes were counted and identified to genus level using a Zeiss Primovert inverted microscope (Carl Zeiss, Inc., White Plains, NY). References consulted for nematode identification include Bongers (1988), Andrássy (2005), and Nickle (1970). Trophic assignments were made with reference to Yeates et al. (1993).

### 2.5 Nematode responses to soil amendment with water and organic matter

Approximately 7 kg of soil were collected from shrubs and interplant spaces at two sites (B, H) for use in a soil amendment experiment. These two sites were selected because they represented a soil that experienced frequent fog and rare rainfall (B) and soil that rarely experienced fog but received more rainfall (H). Soils were sieved (2 mm) and mixed prior to distribution to 250‐ml plastic containers that had been surface sterilized with 70% ethanol. Each of the four soil types (B‐Shrub, B‐Interplant, H‐Shrub, and H‐Interplant) was distributed to containers (300 g each), which were assigned the following four treatments: control, dung‐amended, water‐amended, and dung‐ and water‐amended (*n* = 4 replicates per soil type for a total of 64 experimental units). Dung amendment was accomplished with the application of 5‐g Hartmann's mountain zebra (*Equus zebra hartmannae*) dung that was collected from the gravel plains. The dung was desiccated at the time of collection, broken up manually to pass through a 2‐mm sieve and sterilized by autoclave. Nematodes and other life forms do not survive autoclaving. Dung amendment raised the organic content of shrub soils from 1.16% to 1.91% and of interplant soils from 0.66% to 1.49%. Water amendment was accomplished through the application of 25‐ml sterile, demineralized water via pipette. This treatment raised the moisture content of shrub soils from 2.07 to 9.31 g 100 g^−1^ and of interplant soils from 1.49 to 8.42 g 100 g^−1^. After the application of amendments, each container of soil was mixed with a sterile spatula. Containers were covered loosely with a foil cover and incubated in the dark at room temperature for 30 days. Under field conditions, soils do not remain moist for this length of time following rainfall, nor do they receive large pulses of organic matter (and dung chemistry is different than plant litter chemistry). The purpose of this experiment was not to mimic field conditions, however, and the treatment levels were simply designed to remove resource limitations to investigate the relative potential for moisture and organic matter to influence soil nematode abundance through the decomposer food web. Within 24–48 h of the conclusion of the experiment, nematodes were extracted from soils. Soil properties were measured as described above.

### 2.6 Nematode response to fog

Whether nematodes are anhydrobiotic or active can be determined by examining their morphology after extraction from soils using sucrose solutions (Demeure et al., 1979). Anhydrobiotic nematodes coil their bodies (Figure 7d). Soil samples were collected before, during, and after a fog event from shrub soils at site B on 27–28 September 2020. Fog events are unpredictable and challenging to study in situ, which necessitated limiting this experiment to a single field site. First, soils were collected from five shrubs at the site as described above for routine characterization of nematode abundance and diversity. Then, additional samples were collected over time from other, nearby shrubs to determine the anhydrobiotic status of the nematodes. Different shrubs were used to minimize disturbance at the site. These soils were not sieved, but 50 g of soil collected from each shrub were immediately transferred to 100 ml 1.25 M‐sucrose solution to fix the nematodes in the activity state they were in at the exact time of sampling (anhydrobiotic or active). Five soils were sampled and sucrose‐fixed at 20:00, 08:00, and 12:00. Sucrose‐fixed samples were stored in coolers until returning to the laboratory at 14:00, and then they were refrigerated.

Within 24–48 h, each of the sucrose‐fixed samples was subjected to a sieving procedure to isolate nematodes from the soil material (Treonis et al., 2000). Sucrose solutions were used throughout this procedure to avoid exposing nematodes to pure water, which would have induced uncoiling. Soils and sucrose were mixed in the collection jar and then poured onto stacked 425‐ and 38‐μm sieves. Sucrose (1.25 M) was used to rinse the material retained on the top sieve. Then, more sucrose was used to rinse the material retained on the bottom sieve to remove as many soil particles as possible. The soil retained was rinsed into a beaker. Finally, to separate nematodes from silt particles, the solution in the beaker was slowly transferred with a 5‐ml pipette into a 50‐ml conical tube of cold 2M sucrose solution (2°C). These tubes were allowed to rest (5°C) for 5 h, during which heavier particles settled to the bottom of the tube while lighter material (including nematodes) was retained above. This top layer was poured onto a 25‐μm sieve, and the material retained on the sieve was transferred to a dish for microscopic analysis. The morphology of each nematode encountered was classified as either uncoiled (active or dead at the time of sampling) or coiled in a full circle or whorl (anhydrobiotic and inactive at the time of sampling).

### 2.7 Statistical analyses

Statistical analyses were performed with R version 4.1.0 (https://www.r‐project.org) (R Development Core Team, 2021). Elevation increased with distance from the coast (Table 1), allowing this variable to serve as a proxy for the fog gradient. Correlations between elevation, soil properties, nematode abundance, taxonomic richness, and the Gini–Simpson Index were tested using the Kendall rank correlation coefficient due to non‐normal distributions for many variables. For the soil amendment experiment, ANOVA was used to investigate differences in ln(*x* + 1)‐transformed nematode abundance in response to water and/or dung, with soil type as a fixed effect. The Shapiro–Wilk test was used to assess the normality of the data following transformation. Where treatment effects were significant, means were compared using the Tukey HSD multiple comparison procedure. The proportion of nematodes uncoiled in samples collected over a fog event was ln‐transformed, and the three time points were compared by one‐way ANOVA, followed by Tukey's HSD. A constrained linear canonical ordination (RDA, Rao, 1964) was performed using the R vegan package (Oksanen et al., 2018) to test the contribution of measured soil properties to the abundance of nematodes from each taxa represented, using only nematode types found in more than one sample. Variables included soil moisture, organic content, pH, electrical conductivity, and whether the sample was from a shrub or interplant space. The abundances of each nematode taxon were Hellinger‐transformed prior to RDA analysis.

## 3 RESULTS

### 3.1 Gravel plains nematode communities across the fog gradient

Meteorological data (2017–2020) show that fog decreased from west to east, moving inland from the coast (Figure 2a). Averaged monthly totals for fog precipitation were an order of magnitude greater than rainfall at the two meteorological stations closer to the coast, and rain precipitation was slightly greater than fog at the meteorological station furthest inland (Figure 2a,b). While the averages across the time period highlight the overall variation in fog and rain moisture along the gradient, it is important to note that on most days, soils across the gradient do not experience any fog or rain.

**FIGURE 3:**
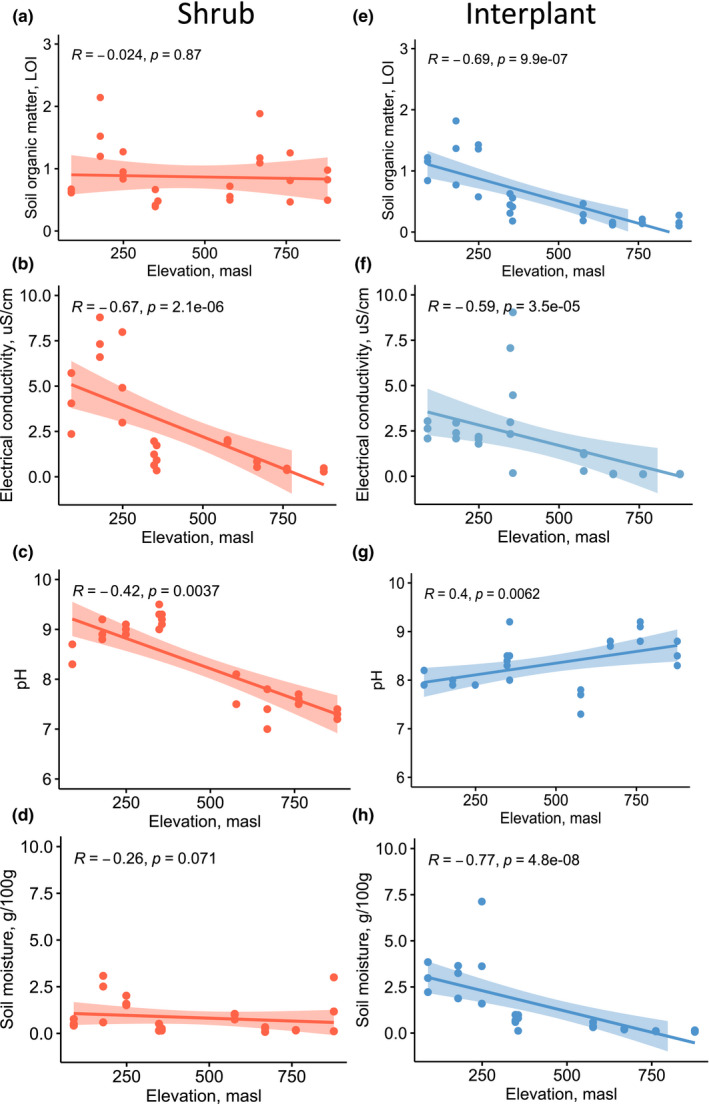
Correlations between elevation and soil properties in shrub and interplant soils collected along a fog gradient in the Namib Desert gravel plains. The elevation is a proxy variable for the fog gradient, as fog declines with increasing elevation. The shaded area represents a 5% confidence interval of Kendall's rank correlation coefficient (R, *n* = 54). Masl = meters above sea level

Elevation was used as a proxy variable for the fog gradient, as elevation increased moving inland from the coast, correlating to decreasing fog frequency. Soil properties varied as elevation increased, but for most variables the pattern was different for shrub and interplant soils (Figure 3). The organic matter did not vary with elevation in shrub soils (Kendall's rank correlation test, *p* = .87), but it decreased with a gain in elevation in interplant soils (Kendall's rank correlation test, *p* < .0001, Figure 3a,e). Soil organic matter was higher in soils under shrubs than in interplant soils (0.87% ± 0.087 vs. 0.57% ± 0.096, *t*‐test, *p* = .001). Soil electrical conductivity declined with increasing elevation in shrub and interplant soils (Kendall's rank correlation test, *p* < .001 for both, Figure 3b,f). Soil pH declined in soils under shrubs (Kendall's rank correlation test, *p* = .0037) and increased in interplant soils (Kendall's rank correlation test, *p* = .0062), as elevation increased (Figure 3c,g). Finally, soil moisture did not change with elevation in shrub soils (Kendall's rank correlation test, *p* = .071), but declined in interplant soils (Kendall's rank correlation test, *p* < .001, Figure 3d,h). Overall, shrub soils had a similar organic matter and moisture contents across the fog gradient, but pH decreased, which may reflect differences in shrub litter chemistry between the two species found at each end of the gradient. Interplant soil organic matter declined across the gradient, which may reflect a reduction in fog‐supported biological crust productivity and/or an increased decomposition rate. All soils showed declining electrical conductivity as elevation increased, which may reflect the influence of more frequent rainfall on salt mobility.

The 14 nematode genera found in the gravel plains soils were all microbivorous. The bacterial‐feeding genera present were *Acrobeles*, *Chiloplacus, Drilocephalobus*, *Elaphonema*, *Mesorhabditis*, *Nothacrobeles*, *Panagrolaimus*, *Panagrobelus*, *Paracrobeles*, and *Zeldia*. The fungal‐feeding genera present were *Aphelenchoides*, *Aphelenchus*, *Hexatylus*, and *Nothotylenchus*. *Panagrolaimus* was the most common nematode found (60% of all nematodes). Of the 54 soil samples collected from the fog gradient, 51 contained nematodes (94.4%, range 0–19,250/kg).

**FIGURE 4:**
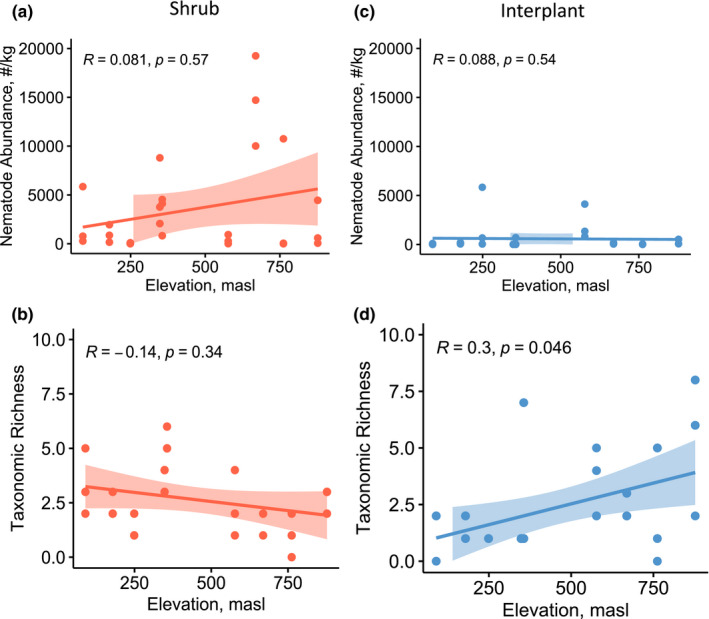
Correlations between elevation and nematode abundance and richness in shrub and interplant soils. The elevation is a proxy variable for the fog gradient, as fog declines with increasing elevation. The shaded area represents a 5% confidence interval of Kendall's rank correlation coefficient (R, *n* = 54). Masl = meters above sea level

Nematode abundance was not correlated to any measured soil properties in shrub or interplant soils (moisture, organic matter, pH, or EC, Kendall's rank correlation test, *p* > 0.1 for each). Nematode abundance did not vary with elevation, that is, across the fog gradient, in either shrub or interplant soils (Kendall's rank correlation tests, *p* > 0.1 for each, Figure 4a,c). Nematode richness ranged from 0 to 8 genera per sample (mean = 2.5). Richness increased in interplant soils as elevation increased (Kendall's rank correlation test, *p* = .046, Figure 4D), while shrub soils showed no change (Kendall's rank correlation test, *p* = .34, Figure 4b). Counterintuitively, as nematode richness increased in interplant soils (Figure 4d), soil organic matter content and moisture content decreased (Figure 3e,h). The Gini–Simpson Index showed the same pattern as richness (data not shown).

**FIGURE 5:**
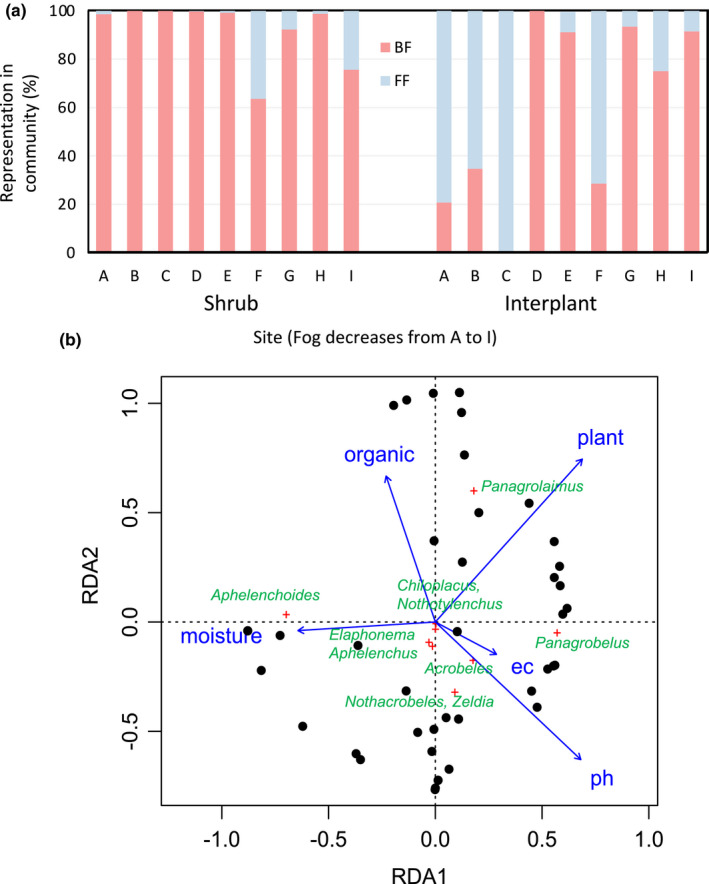
(a) Proportional representation of bacterial‐feeders (BF) and fungal‐feeders (FF) within nematode communities in soils collected under shrubs or interplant spaces. (b) Ordination biplot for RDA of relationships among soil properties and the abundance of nematode taxa in gravel plains soils (*n* = 51 soils that contained nematodes, ec = electrical conductivity, organic = organic matter content, moisture = moisture content, plant = soil from under a shrub, ph = pH)

At all sites, shrub nematode communities were dominated by bacterial‐feeding taxa (Figure 5a). Interplant nematode communities at sites A, B, C, and F were dominated by fungal‐feeding taxa while other interplant sites were dominated by bacterial‐feeders (Figure 5a). Soil properties were associated with specific nematode taxa (RDA, Figure 5b). RDA found that the first two axes explained 23.9% of the variation in nematode community trophic structure (Figure 5b). Monte Carlo permutation testing (*n* = 999) determined the axes explained a significant portion of the variance in the dataset (*p* = .001). *Panagrolaimus* was associated with shrub soils and higher soil organic matter content, and *Aphelenchoides* was associated with higher soil moisture levels (Figure 5b). *Panagrobelus* was associated with shrub soils and higher electrical conductivity and pH (Figure 5b). *Nothacrobeles, Zeldia, and Acrobeles* were associated with higher pH (Figure 5b). Other nematode taxa did not show relationships to soil variables (Figure 5b). RDA only explained 23.9% of the variation in the nematode communities, suggesting that there are other unmeasured variables affecting the distribution and abundance of nematode taxa in gravel plains soils.

### 3.2 Nematode communities within soil microhabitats

Soils collected at site B to investigate nematode density within microhabitats found that most nematodes were located in the 1–5 cm layer of soil (mean = 156.3/kg) versus the surface 0–1 cm (mean = 10.4/kg). Rocks and biological crust material that was retained on a 2‐mm sieve from the 0–1 cm layer also contained few nematodes (mean = 31.3/kg), suggesting that sieving had a minor effect on our estimates of nematode abundance in soils that had significant crusts (interplant soils at sites A and B only). Hypolithic soils under quartz rocks contained nematodes in abundances similar to interplant soils, and the communities consisted almost entirely of *Aphelenchoides* (range 0–4000/kg, mean = 459.7). Lichen samples did not yield any nematodes.

### 3.3 Nematode response to soil amendment with water and organic matter

**FIGURE 6:**
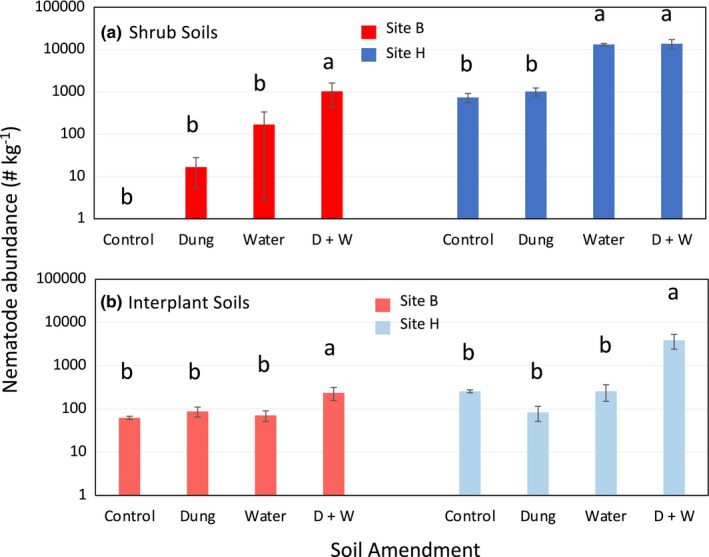
Nematode abundance in (a) shrub and (b) interplant soils at the conclusion of a 30‐day soil amendment experiment for which dung and water (alone or in combination) were added to gravel plains soils from two sites (B, H). Values are means ± the standard error of the mean (*n* = 4 samples/bar). Within a site*soil type grouping, lower case letters indicate significant differences (Tukey's HSD test)

Nematodes responded to soil amendment (ANOVA, significant site*treatment effect, *p* < .001). In shrub soils from site B and interplant soils from sites B and H, only dung + water amendment increased nematode abundance significantly (Figure 6a,b). Water alone increased nematode abundance in shrub soils from site H (Figure 6a).

Although nematodes responded to amendment, the nematode taxa that contributed to this response were distinct among the soil types. In soils from site B, only bacterial‐feeding taxa increased in dung + water amended soils (*Chiloplacus* in interplant soils vs. *Panagrolaimus* in shrub soils). However, in soils from site H, a more diverse suite of fungal‐ and bacterial‐feeding taxa increased (*Nothacrobeles*, *Zeldia*, *Aphelenchus*, *Aphelenchoides*, and *Hexatylus* in interplant soils and *Panagrolaimus*, *Mesorhabditis*, and *Aphelenchoides* for shrub soils).

### 3.4 Nematode response to fog

**FIGURE 7:**
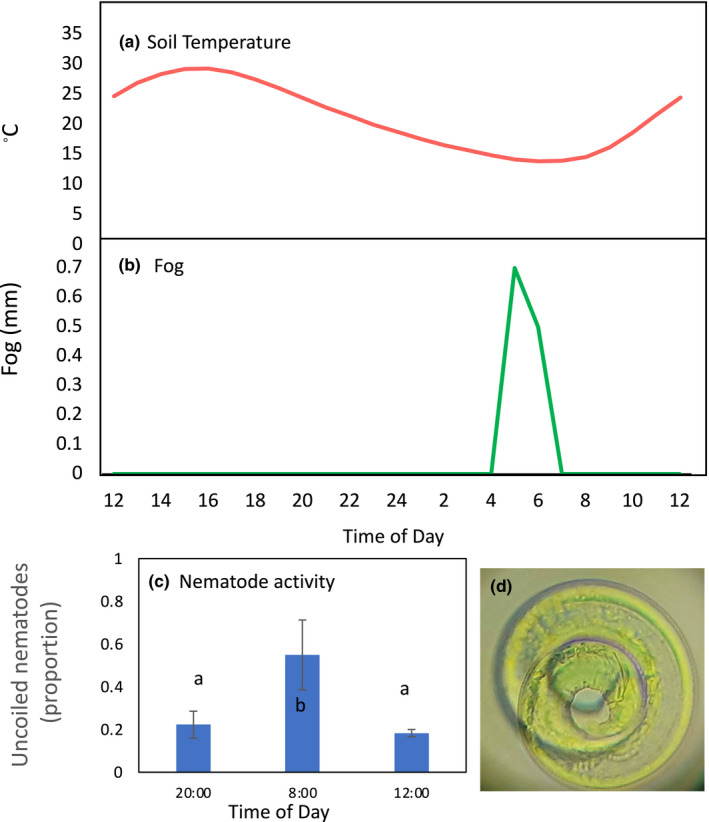
(a) Soil temperature (3‐cm depth), (b) fog, and (c) nematode activity over a fog event in gravel plains soils. For (c) values are means ± the standard error of the mean. Lower case letters indicate significant differences among the times of day (Tukey's HSD test). (d) Anhydrobiotic (i.e., coiled) nematode (uncoiled body length is approximately 700 μm)

Over a 16‐hr period at site B, soil temperature and fog moisture varied (Figure 7a,b). From 04:00 to 07:30 a moderate fog event occurred. Soil moisture content increased from 1.07 g 100 g^−1^ at 20:00 to 2.76 at 08:00, and then dropped to 1.43 g 100 g^−1^ at 12:00. The proportion of nematodes that were uncoiled was highest at 08:00 (0.55), immediately following the fog event, as compared to 20:00 or 12:00 (0.22 and 0.18, respectively, ANOVA, *p* = .044, Figure 7c). *Panagrobelus* was the only nematode found in these samples.

## 4 DISCUSSION

Nematodes are ubiquitous in the hyper‐arid, gravel plains soils of the Namib Desert, which is likely tied to their use of the desiccation survival strategy, anhydrobiosis. We found that nematodes rapidly emerged from anhydrobiosis in response to soil wetting during a fog event. This study is the first to demonstrate that moisture from fog can stimulate nematode activity in soils. While only one nematode species was present in the samples analyzed (*Panagrobelus*), the use of anhydrobiosis has been established for a wide variety of nematode taxa, including several taxa abundant in the gravel plains soils [e.g., Panagrolaimidae, *Aphelenchus,* Cephalobidae; Treonis & Wall, 2005]. The use of anhydrobiosis by gravel plains nematodes may be a critical aspect of their physiology that allows for their wide distribution in this environment where the type and frequency of precipitation are highly variable.

Our meteorological data are consistent with prior, more extensive analyses documenting that the prevalence of fog declines in the Namib Desert with increasing elevation, moving inland from the Atlantic Coast, creating a well‐delineated fog gradient (Spirig et al., 2019). Prior research has shown that fog can raise the moisture content of gravel plains soils (Li et al., 2018; Wang et al., 2019). However, Li et al. (2018) also failed to detect a correlation between fog and soil moisture at one gravel plains field site over an 80‐day period, suggesting the relationship could be more stochastic. Unfortunately, we were not able to directly measure soil moisture in response to fog at each of our field sites due to the unpredictable nature of fog and the lack of access to deployable instrumentation. We did measure a significant increase in soil moisture during a modest fog event at site B. Although the soil texture was similar across our field sites, it is possible that the penetrability of fog moisture into soil varies among our field sites due to topography, the presence of desert varnish, or the nature of the fog itself (e.g., droplet size, duration, etc.). More research would be valuable to establish the direct impact of fog on soil moisture across the Namib Desert gravel plains, but evidence exists to support the general likelihood that fog events can raise soil moisture.

Because fog can trigger nematode emergence from anhydrobiosis, creating a favorable environment for nematode feeding and reproduction, we predicted that more frequent fog‐derived moisture inputs near the coast would promote nematode abundance and diversity. Contrary to our prediction, we found no effect of position in the fog gradient on soil nematode abundance. Armstrong et al. (2016) monitored the structure of bacterial communities over 12 months at a single site in the fog gradient of the gravel plains. In this study, microbial communities did not vary among the time points, except immediately following a significant rainfall, suggesting that fog events over the study period either failed to wet the soil or were not sufficient to impact bacterial diversity. Regardless of whether fog consistently moistens the gravel plains soils across the gradient or not, our results from soils in the field align with most of the results from the soil amendment experiment where nematodes generally did not respond numerically to amendment with water alone.

Nematodes in soils from other deserts have been shown to be non‐responsive to variation in soil moisture, either through experimental amendment or from natural sources. For example, Vandegehuchte et al. (2015) found that microbivorous nematodes did not respond to 4 years of simulated rainfall in Chihuahuan Desert soils. Similarly, Simmons et al. (2009) and Treonis et al. (2002) found that moisture addition did not affect nematode density in the Antarctic Dry Valleys. Other studies have found that desert nematode abundance was not enhanced in areas of ephemeral water flows (i.e., rainfall or snowmelt channels, Treonis et al., 1999; Treonis et al., 2019). In contrast, Zawierucha et al. (2019) found that the Antarctic nematode *Scottnema lindsayae* increased in abundance with elevation, possibly responding to soil moisture derived from clouds, and Steinberger and Sarig (1993) documented increased nematodes following wetting of soils in the Negev Desert. More work is needed to understand the interplay among fog, soil moisture, and nematode abundance in arid ecosystems. In the Namib Desert gravel plains, the fog gradient we studied may not induce biologically significant variation in soil moisture, either because the soil is not wetted sufficient or because water is not the primary factor limiting nematode abundance in these soils.

When gravel plains soils were subject to co‐amendment with moisture and organic matter for 30 days in vitro, most nematode communities were numerically responsive. No changes were found in soils amended with organic matter alone, and nematodes responded to moisture amendment alone in only one of the four soil types tested. The treatments and duration of exposure for the soil amendment experiment were different from what these nematodes are likely to experience in the field, where organic matter and moisture are more likely to be available in shorter pulses. However, the amendment experiment demonstrates the sensitivity of the community to both of these factors. The results suggest that organic matter is a key factor limiting nematode abundance in gravel plains soils and that they can only take advantage of increased availability of this resource if moisture also is available to support their activity. However, these results are inconsistent with the lack of variation in nematode abundance across the landscape. Moisture and organic matter differed naturally among sites, but these variables were not correlated to abundance. In the field, the short duration of moisture pulses may limit the capacity for nematodes to respond to variation in organic matter.

Soil nematode abundance in deserts is thought to be closely tied to soil organic matter content, which supplies substrate to the decomposer microorganisms (bacterial and fungi) that many nematodes consume for energy (Treonis et al., 2019). Nematodes in the gravel plains of Namibia may be an exception to this trend, despite demonstrating organic matter limitations in the soil incubation experiment. Soil organic matter was higher in soils associated with shrubs, but nematode abundance was comparable to interplant soils for most samples. This contrasts with the higher nematode abundance Marais et al. (2020) measured in the Namib Desert sand dunes under *A. horridus* shrubs, compared to interplant soils. In the gravel plains, however, there were visible biological soil crusts in interplant spaces at the sites closest to the coast, in the “lichen fields” of the Namib Desert (sites A, B, and C). Like nematodes, lichens have also been shown to activate metabolically in response to fog in the Namib Desert (Lange et al., 1994). Biological soil crusts, consisting of fungi, lichens, cyanobacteria, and other microorganisms, occupy much of the soil surface area in arid and semi‐arid ecosystems (Pointing & Belnap, 2012 **)**. Crusts likely were present in all the interplant spaces we sampled, although only visible at sites A‐C, where fog is frequent. Soil organic matter was not high in interplant soils, but the quality of the material is likely different than organic matter formed from shrub litter, and it may be highly suitable for supporting nematodes and their microbial food sources. Fungal‐feeding nematodes were particularly abundant in interplant soils at sites A, B, C, and F, as well as in the hypolithic soils sampled from sites A–E. Some of these soils had higher soil moisture, perhaps due to recent fog events, and *Aphelenchoides*, a fungal feeder, was found by RDA to be associated with higher moisture. The interplant spaces in the fog‐fed, lichen fields of the Namib Desert may represent a unique habitat for nematodes that subsist on fungi from the crusts.

Although nematode abundance did not vary across the fog gradient, our data suggest that nematode diversity was highest where the fog was least frequent, specifically in interplant soils, and again, contrary to predictions. Soils at the eastern and most inland part of the fog gradient were drier and contained less organic matter than soils nearest to the coast. They were also less saline (lower electrical conductivity), likely because of the effects of more frequent rainfall on salt mobilization. Yet, they contained more diverse nematode communities. During our study period, the gravel plains landscape appeared similar across the fog gradient, with widely spaced perennial shrubs as the only visible vegetation. This landscape changes dramatically, however, following significant rainfall, and although infrequent and unpredictable, rainfall is more frequent further inland (Eckardt et al., 2013). When significant rain does fall in Namib, such as was measured at the Garnet Koppie meteorological station in 2017 and 2018 (Figure 2), the growth of grasses and forbs in intershrub spaces dramatically transforms the landscape. Desert ephemerals such as *Stipagrostis* grasses germinate after at least 20 mm of rainfall (Seely & Pallett, 2008). These plants also attract grazing animals, increasing dung and urine inputs to the soil that may affect soil food webs, as has been seen in other ecosystems (Pan et al., 2022). Furthermore, the composition and function of Namib Desert soil microbial communities have been shown to be dramatically altered by significant water inputs, both in ex situ microcosm experiments (Frossard et al., 2015) and in in situ field studies (Armstrong et al., 2016).

Although we could not study the direct impact of rain on nematodes during the time span of this research, rainfall‐induced vegetation may have a persistent effect on soil nematodes even after the plants are grazed down. Higher nematode diversity in the interplant soils that were most likely to experience rain may be due to the legacy effect of resources created by the ephemeral plants that grew following significant rainfall events. In an unusually rainy year, Hachfeld (2000) found an increase in plant diversity along the fog gradient encompassing our field sites, with the richness of shrubs, grasses, and forbs increasing from 4 to 14 1000 m^−2^, moving from 20 to 110 km inland. Linkages between above‐ and below‐ground biodiversity are challenging to demonstrate but have been seen in other ecosystems (Porazinska et al., 2018). Furthermore, in the Mojave Desert, a rainfall‐induced “superbloom” of desert annuals was associated with a rapid increase in nematode abundance in intershrub soils (Treonis et al., 2019). Ephemeral plants appear to have an important role in supporting nematode biodiversity in desert soils. Other studies have shown variation in biological assemblages across the fog gradient in the Namib Desert, including collembola, ants, and bacteria. Species richness for these organisms also increased moving inland, particularly when the sampling effort is extended farther and into areas that receive rain more often (Collins et al., 2019; Marsh, 1986; Scola et al., 2018).

Specific nematode taxa were linked to shrubs throughout the gravel plains. RDA showed that *Panagrolaimus* was specifically associated with shrubs and soils with higher organic matter content. These nematodes were found throughout the fog gradient. *Panagrobelus* was also abundant under shrubs but only the higher salinity *A. leubnitziae* shrubs at the sites nearest to the coast (sites A–E). This nematode was not present in shrub soils under *P. leubnitziae*. The two shrub species that were sampled across the fog gradient are very different, *A. leubnitziae* is smaller, with fleshy stems and small, scale‐like leaves with relatively little plant litter. In contrast*, P. leubnitziae* had loose but deep layers of dropped leaf litter (2–10 cm). *Panagrolaimus* and *Panagrobelus* were rare in interplant soils, and these nematodes may be specialists for the relatively organic‐rich environment under perennial plants in the Namib Desert, although decomposition rates are likely to be very slow. *Panagrobelus* seems to be better adapted to *A. leubnitziae* shrubs in the area of the desert with frequent fog, which may relate to its ability to respond to fog‐induced changes in soil moisture by emerging from anhydrobiosis.

Our study provides insights into how fog influences soil nematode diversity and abundance across the Namib Desert gravel plains. Soil nematodes appear to be well‐adapted to varying resource availability and soil functioning across the fog gradient. A combination of organic matter from multiple sources, including biological soil crusts, ephemeral vegetation, and perennial shrubs, seems to be supporting nematode communities, indicative of the diverse soil functioning within this system. Nematodes responded to a fog event that wetted the soil, allowing them to emerge from anhydrobiosis and become active. Fog appears to be an important resource that sustains biological soil crusts, microbes, and microbivorous nematodes in many gravel plains soils. In contrast, in areas that rarely experience fog, sporadic rainfall events may be generating pulses of ephemeral plant productivity and diversity that sustain higher diversity nematode communities. The gravel plains of the Namib Desert are experiencing several anthropogenic threats. Mining and tourism affect the physical integrity of the soil, with mining an intense, but highly localized activity (Wassenaar et al., 2013), while tourism impacts (i.e., mainly off‐road driving) are widespread and more diffuse. Furthermore, there is concern about the effects of a changing global climate on the frequency and distribution of precipitation in the Namib Desert (Rohde et al., 2019). Temporal monitoring of gravel plains vegetation and nematode communities could be useful for assessing the impacts of land use and climate change on this unique ecosystem where organisms are already existing at the margins of habitability.

## 5 AUTHOR CONTRIBUTIONS

**Amy M. Treonis:** Conceptualization (lead); data curation (lead); formal analysis (lead); funding acquisition (lead); investigation (lead); methodology (lead); resources (equal); writing – original draft (lead); writing – review and editing (lead). **Eugene Marais:** Conceptualization (supporting); investigation (supporting); methodology (supporting); resources (equal); writing – original draft (supporting); writing – review and editing (supporting). **Gillian Maggs‐Kölling:** Conceptualization (supporting); resources (equal); writing – original draft (supporting); writing – review and editing (supporting).

## 7 CONFLICT OF INTEREST

The authors have no conflicts of interest to declare.

## Data Availability

Data is available from the Dryad Digital Repository: https://doi.org/10.5061/dryad.pk0p2ngqg
